# Early postoperative mortality similar between cemented and uncemented hip arthroplasty: a register study based on Finnish national data

**DOI:** 10.1080/17453674.2018.1558500

**Published:** 2019-02-04

**Authors:** Elina Ekman, Antton Palomäki, Inari Laaksonen, Mikko Peltola, Unto Häkkinen, Keijo Mäkelä

**Affiliations:** a Department of Orthopaedics and Traumatology, Turku University Hospital, Turku, Finland;; b National Institute for Health and Welfare, Helsinki, Finland

## Abstract

Background and purpose — Implant survival of cemented total hip arthroplasty (THA) in elderly patients is higher than that of uncemented THA. However, a higher mortality rate in patients undergoing cemented THA compared with uncemented or hybrid THA has been reported. We assessed whether cemented fixation increases peri- or early postoperative mortality compared with uncemented and hybrid THA.

Patients and methods — Patients with osteoarthritis who received a primary THA in Finland between 1998 and 2013 were identified from the PERFECT database of the National Institute for Health and Welfare in Finland. Definitive data on fixation method and comorbidities were available for 62,221 THAs. Mortality adjusted for fixation method, sex, age group, and comorbidities among the cemented, uncemented, and hybrid THA was examined using logistic regression analysis. Reasons for cardiovascular death within 90 days since the index procedure were extracted from the national Causes of Death Statistics and assessed separately.

Results — 1- to 2-day adjusted mortality after cemented THA was comparable to that of the uncemented THA group (OR 1.2; 95% CI 0.24–6.5). 3- to 10-day mortality in the cemented THA group was comparable to that in the uncemented THA group (OR 0.54; CI 0.26–1.1), and in the hybrid THA group (OR 0.64, CI 0.25–1.6). Pulmonary embolism or cardiovascular reasons as a cause of death were not over-represented in the cemented THA group.

Interpretation — Early peri- and postoperative mortality in the cemented THA group was similar compared with that of the hybrid and uncemented groups.

The early postoperative mortality after total hip arthroplasty (THA) is low and has been decreasing over the last few years (Aynardi et al. 2009, McMinn et al. 2012, Lalmohamed et al. 2014). 2 recent publications have indicated that 90-day mortality after primary THA performed for any indication is 0.7% (Hunt et al. 2013, Garland et al. 2015). Improvements in surgical techniques and implants, the introduction of low molecular weight heparins in the 1980s, and operative room sterility have significantly reduced mortality risks (Nurmohamed et al. 1992, Harris 2009). On the other hand, the surgery is now being performed on older patients who often have multiple comorbidities, which increase adverse outcomes (Mahomed et al. 2003, Bozic et al. 2012).

Cementing has been used for decades for THA implant fixation with good implant survival rates in long term follow-up (Morshed et al. 2007, Mäkelä et al. 2014a). However, cemented fixation is associated with potential perioperative morbidity in the form of bone cement implantation syndrome where fat and bone marrow cause emboli during cement pressurizing into the pulmonary arteries and may lead to intra- or early postoperative hypotension, and even death of the patient (Donaldson et al. 2009). Even though survival of cemented implants in the elderly population is higher than the survival of uncemented implants, the fear of bone cement implantation syndrome is one of the reasons for the increased use of uncemented implants (Junnila et al. 2016). The leading cause of death after THA is cardiovascular (Berstock et al. 2014).

We studied whether early postoperative mortality of patients treated with uncemented THA differed from that of patients treated with cemented or hybrid THA based on data from the PERFECT database maintained by the National Institute for Health and Welfare in Finland. We also assessed bone cement implantation syndrome and early cardiovascular mortality in this same population.

## Patients and methods

### Study population

The study population was identified from the Finnish Hospital Discharge Register (FHDR) using the 10th revision of the International Classification of Diseases (ICD-10) diagnosis codes M16.0 to M16.9, and the Finnish version of NOMESCO Classification Procedural Codes NFB30 (uncemented THA), NFB40 (hybrid THA when only the femoral stem has been cemented), or NFB50 (cemented THA). During the study period from January 1, 1998 to December 31, 2013, 73,915 patients were treated with THA for primary or secondary OA in Finland. Definitive data on fixation method and comorbidities were available for 62,221 THAs, which formed the final study population.

All public and private hospitals in Finland are obliged to report all surgical procedures to the Finnish National Institute of Health and Welfare. The present study was based on the PERFECT (PERFormance, Efficiency, and Costs of Treatment Episodes) hip replacement database, which uses data from numerous registries such as the Hospital Discharge Register (maintained by the Finnish National Institute of Health and Welfare), cause of death statistics maintained by Statistics Finland, the Social Insurance Institution’s drug prescription register and drug reimbursement register, and the Finnish Arthroplasty Register. Data on comorbidities, on the use of residential care, patient ID number, provider ID number(s), age, sex, residential area codes, diagnosis, operation codes, date of admission, operation, and the date of discharge or death, whichever came first, were extracted from the PERFECT database (Peltola et al. 2011) (Table 1).

**Table 1.** Background characteristics of patients. Values are frequency (percentage) unless otherwise stated

Background	Cemented	Uncemented	Hybrid
characteristics	THA	THA	THA
Number of patients	23,636	38,477	11,802
Mean age	73.7	64.9	66.5
Men	8,277 (35)	18,747 (49)	5,636 (48)
Hypertension	11,104 (47)	15,771 (41)	4,725 (40)
Ischemic heart disease	4,234 (20)	4,135 (11)	1,466 (12)
Atrial fibrillation	1,868 (7.9)	2,082 (5.4)	676 (5.7)
Heart insufficiency	1,284 (5.4)	797 (2.1)	431 (3.7)
Diabetes	2,130 (9.0)	3,715 (9.7)	964 (8.2)
COPD and asthma	2,422 (10)	3,973 (10)	1,059 (9.0)
Cancer	1,833 (7.8)	2,575 (6.7)	724 (6.1)
Depression	1,522 (6.4)	2,688 (7.0)	704 (6.0)
Parkinson’s disease	283 (1.2)	381 (1.0)	111 (0.9)
Dementia	281 (1.2)	212 (0.6)	98 (0.8)
Uremia	30 (0.1)	61 (0.2)	19 (0.2)
Mental disorders	659 (2.8)	978 (2.5)	284 (2.4)

These results have not been adjusted via propensity score weighting.

THA = total hip arthroplasty.

The validity of the individual registries mentioned above has been studied. The Finnish Hospital Discharge Register data have been compared with external audit data in 32 studies (Sund 2012). The coverage and positive predictive values have been over 90% in those studies. The prescription database data have been found to be in high concordance with self-reported medication (Haukka et al. 2007).

To assess bone cement implantation syndrome and cardiovascular reasons separately as a cause of death, mortality reported with the associated diagnostic codes (codes I21 acute myocardial infarction, I25 ischemic heart disease, I26 pulmonary embolism, I50 heart failure, and I63 stroke in the ICD-10 classification) within 90 days since the index procedure were extracted from the national Causes of Death Statistics. The validity of the Finnish mortality statistics is reliable (Lahti and Penttilä 2003, Pajunen et al. 2005). The primary outcome used in this study was total mortality and secondary outcome cardiovascular mortality and mortality associated with pulmonary embolism. The patients were followed up for 1 year postoperatively.

### Statistics

Mortality among the cemented, uncemented, and hybrid groups was examined using logistic regression analysis. The analysis was repeated for 365 outcomes that each described the status of the patient (alive/dead) on a certain day after the operation. In order to reduce the effects of confounding in this observational study, differences in distributions of observed covariates between the groups were adjusted: fixation method, sex, age group (< 50, 50–59, 60–69, 70–79, ³ 80), comorbidities (Table 1), and the year of operation. In the model, treatment assignment (cemented/uncemented/hybrid) was the dependent variable and all observed background variables (Table 1) were independent variables, as the aim was to balance all observed covariates between the groups. 95% confidence intervals (CI) were calculated for adjusted mortality.

### Ethics, funding, and potential conflicts of interest

The ethics committee of the Finnish National Institute for Health and Welfare (THL) approved the study (Dnro THL/127/5.05.00/2015). This research received no specific grant from any funding agency. The authors declare no conflicts of interest.

## Results

**Figure 1.**
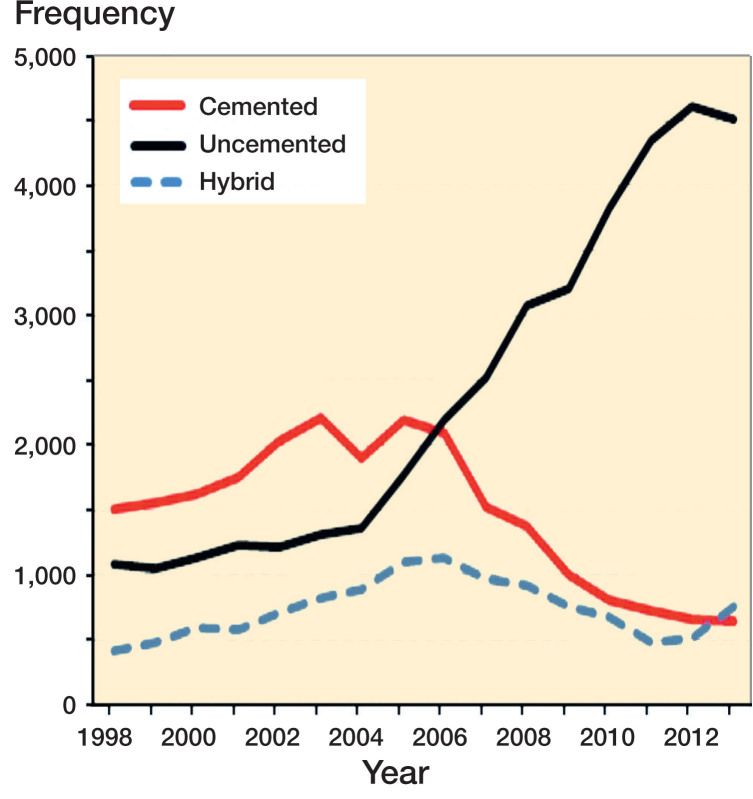
Annual numbers of cemented, uncemented, and hybrid THA in Finland during the study period.

**Figure 2.**
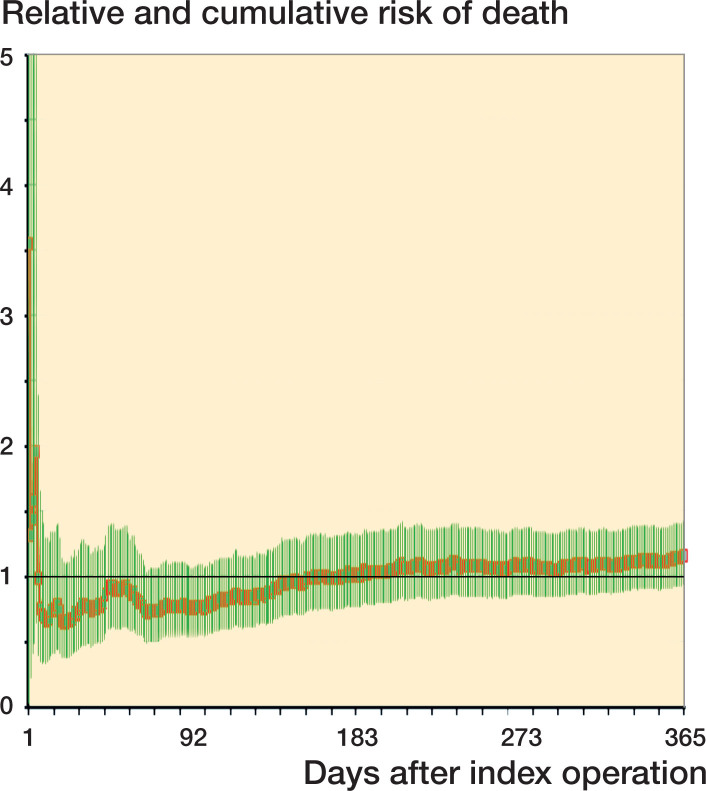
Relative and cumulative risk of death in patients receiving a cemented THA compared with patients receiving an uncemented THA. No statistically significant difference in mortality was found.

The use of cemented THA decreased in Finland during the study period, whereas the use of uncemented THA increased (Figure 1). The adjusted overall mortality or mortality associated with cardiovascular reasons or pulmonary embolism were similar between cemented THA and uncemented or hybrid THA at any of the studied time points (Figure 2). There were 9 deaths during days 1 and 2 in the cemented THA group, 4 in the uncemented THA group, and 0 in the hybrid group (Table 2). The 1- and 2-day adjusted mortality in the cemented THA group was the same as that in the uncemented THA group (OR = 1.2; CI 0.2–6.5) (Table 3).

**Table 2.** Patient mortality, raw data. Values are frequency (percentage)

	Cemented	Uncemented	Hybrid
	THA	THA	THA
Mortality	n = 23,636	n = 38,477	n = 11,802
1–2 days	9 (0.0)	4 (0.0)	0 (0.0)
3–10 days	45 (0.2)	23 (0.1)	6 (0.1)
11–20 days	35 (0.1)	14 (0.0)	6 (0.1)
21–30 days	22 (0.1)	4 (0.0)	3 (0.0)
30 days	111 (0.5)	45 (0.1)	15 (0.1)
90 days	228 (1.0)	93 (0.2)	29 (0.2)
180 days	389 (1.6)	155 (0.4)	52 (0.4)
365 days	712 (3.0)	254 (0.7)	115 (1.0)

**Table 3.** Postoperative mortality risk (OR (95% CI)) for cemented and hybrid THA compared with uncemented THA (reference)

	Cemented THA	Hybrid THA
2 days	1.2 (0.2–6.5)	0 (0.0–999.9)
3–10 days	0.5 (0.3–1.1)	0.6 (0.3–1.6)
11–20 days	0.7 (0.3–1.8)	0.9 (0.3–2.5)
21–30 days	2.8 (0.8–10.0)	1.9 (0.4–8.8)
30 days	0.8 (0.5–1.3)	0.8 (0.4–1.4)
90 days	0.8 (0.6–1.1)	0.7 (0.5–1.1)
365 days	1.2 (1.0–1.4)	1.2 (0.9–1.5)

There were 45 deaths during the days 3 to 10 in the cemented THA group, 23 in the uncemented THA group, and 6 in the hybrid group (Table 2). The 3- to 10-day adjusted mortality in the cemented THA group was similar to that in the uncemented THA group (OR = 0.5; CI 0.3–1.1), and in the hybrid THA group (OR = 0.6, CI 0.3–1.6) (Table 3).

Data on deaths and mortality for the follow-up periods 11 to 20 days, 21 to 30 days, 30 days, 90 days, and 365 days are presented in Tables 2 and 3.

There were no deaths due to pulmonary embolism during days 1 and 2 in any of the groups (Table 4). There were 5 deaths during days 1 and 2 in the cemented THA group due to cardiovascular diseases, 4 in the uncemented THA group, and 0 in the hybrid group (Table 4).

**Table 4.** Causes of death. Values are frequency (percentage)

	Cemented	Uncemented	Hybrid
	THA	THA	THA
Cause of death	n = 23,636	n = 38,477	n = 11,802
1–2 days			
Pulmonary embolism	0	0	0
Other cardiovascular	5 (0.02)	4 (0.01)	0
3–10 days			
Pulmonary embolism	1 (0.00)	2 (0.01)	0
All cardiovascular	11 (0.05)	19 (0.05)	4 (0.03)
11–20 days			
Pulmonary embolism	5 (0.02)	1 (0.00)	1 (0.01)
All cardiovascular	15 (0.06)	9 (0.02)	5 (0.04)
21–30 days			
Pulmonary embolism	3 (0.01)	1 (0.00)	0 (0.00)
All cardiovascular	8 (0.03)	3 (0.01)	2 (0.02)
90 days			
Pulmonary embolism	15 (0.06)	14 (0.04)	4 (0.03)
All cardiovascular	76 (0.32)	60 (0.16)	22 (0.19)
365 days			
Pulmonary embolism	30 (0.13)	20 (0.05)	7 (0.06)
All cardiovascular	208 (0.88)	127 (0.33)	62 (0.53)

All cardiovascular: acute myocardial infarction, ischemic heart disease, pulmonary embolism, heart failure, stroke. Follow-up of the causes of death is to the end of 2013.

Data on cause of death for the follow-up periods 11 to 20 days, 21 to 30 days, 30 days, 90 days, and 365 days are presented in Table 4.

## Discussion

Based on Finnish Registry data the adjusted early postoperative mortality after cemented THA compared with uncemented or hybrid THA was similar as regards death for any reason, death from pulmonary embolism, or death for cardiovascular reasons. However, using unadjusted data the proportion of perioperative deaths was higher in patients with cemented THA than in patients with uncemented or hybrid THA.

Cementing is the gold standard for implant fixation, especially in elderly patients. In combined Nordic data, risk for revision has been both statistically and clinically significantly lower with cemented implants than with uncemented implants in patients aged 65 years or more (Mäkelä et al. 2014a, Varnum et al. 2015). Bone cement has been thought to strengthen bone from inside and therefore to decrease the risk for periprosthetic fracture, osteolysis, and loosening. Lower revision rates for cemented implants in elderly patients have been found in all major registries (Swedish Hip Arthroplasty Register 2013, AOANJRR 2016, NJR 2016). Even though superiority in implant survival of cemented THA in elderly patients, fear of bone cement implantation syndrome (BCIS) has led many surgeons towards using uncemented implant fixation (Dale et al. 2009, Fevang et al. 2010, Mäkelä et al. 2014b). BCIS is characterized by perioperative hypotension and hypoxia, and at worst cardiac arrest and death of the patient. The true incidence of cardiac arrest secondary to BCIS is unknown (Donaldson et al. 2009). In our study the 1- and 2-day adjusted mortality was similar in the cemented and uncemented THA groups. Thus, BCIS is seldom a cause of death in elective THA patients in Finland.

Historically, cemented THA has been associated with greater than 3-fold higher intraoperative mortality (Coventry et al. 1974, Ereth et al. 1992, Parvizi et al. 1999), but at the end of the 1990’s a reduction in the intraoperative mortality rate has been reported (Parvizi et al. 1999) and the mortality has decreased even more during the twenty-first century (Hunt et al. 2013). A Swedish register study reported an increased adjusted risk of death during the first 14 days after surgery in patients who underwent cemented THA when compared with matched controls (HR of 1.3, 95% CI 1.11–1.44). This means 5 additional deaths per 10,000 observations. Such an increased risk of death was not found in patients with a cementless or hybrid THA. However, this risk in the cemented THA group disappeared during follow-up of 90-days (Garland et al. 2017). In our study the adjusted OR for mortality in the cemented THA group was not elevated during the first 20 postoperative days when compared with the uncemented THA group. Also, McMinn et al. (2012) reported a higher mortality rate in patients undergoing cemented THA compared with uncemented THA. However, this increase in mortality occurred gradually during 8 years after surgery and not early as would be expected if the increased mortality was caused by BCIS. We found similar adjusted mortality regarding the use of bone cement at any time point up to 365 days postoperatively. This is in line with a study by Parvizi et al. (2001) who found no increased risk of death with cemented THA 30 days postoperatively.

In a recent systematic review the overall 30-day mortality was 0.30% and 90-day mortality was 0.65% following THA. The leading cause of death was ischemic heart disease (41% of deaths) followed by cerebrovascular accidents (23%), and pulmonary embolism (12%) (Berstock et al. 2014). In our material the unadjusted mortality at 30 and 90 days for the cemented THA group was 0.5% and 1.0%, and 0.1% and 0.2% for the hybrid and uncemented groups, respectively. These differences are mainly explained by patient selection, and after adjusting for the elderly and sicker population in the cemented group the mortality was similar between the cemented and uncemented groups. The leading cause of death was cardiovascular. Parvizi et al. (1999) studied intraoperative mortality during cemented THA and found an incidence of 0.03%, the leading cause of death being pulmonary embolism. In previous studies increasing age, male sex, worse ASA score (> 3), and higher number of comorbidities have been found to increase the risk of death after THA surgery (Bozic et al. 2012, Mahomed et al. 2003, Parvizi et al. 2001, Hunt et al. 2013). In our study we attempted to account for this by adjusting the treatment groups for sex, age, and comorbidities, whereafter early overall mortality between the groups was similar at any time point.

Our study has several limitations. First, we have no information regarding perioperative resuscitations because of cardiac arrest due to BCIS. Second, data on revision surgeries of the study patients were not included. Thus, we do not know whether mortality is associated for example with multiple operations. Third, the number of deaths for cardiovascular accidents or pulmonary embolism in our study was fairly small. It is possible that in a larger population some smaller differences in the mortality could be detected. Nonetheless, our material consisted of over 60,000 THAs and therefore we believe that there is no difference in clinical importance. Further the PERFECT database does not include information on patients’ socioeconomic status, which is known to affect mortality after THA (Whitehouse et al. 2014, Garland et al. 2017). Therefore some amount of residual confounding cannot be ruled out.

As we modeled the mortality difference between the groups using logistic regression analysis repeatedly (356 analyses) there is a possibility of overfitting as the statistical model may contain more parameters than can be justified by the data. This can mean that the results are based on an adaptation to random variation in the sample and therefore the conclusions of our sample could not be generalized to a greater population.

In summary, adjusted perioperative and short-term mortality was similar between patients treated with cemented THA and patients treated with uncemented or hybrid THA. This pertained also when cardiovascular and pulmonary embolism mortality was studied separately. Based on our results and earlier literature, cemented THA is a safe option and should be the gold standard in the elderly patient population. 

KM designed and coordinated the study. EE collected the data and drafted the manuscript. AP helped to draft the manuscript. MP and UH calculated the statistics. All authors contributed to the interpretation of the data and results and to the preparation of the manuscript.

*Acta* thanks Ross W Crawford for help with peer review of this study

AOANJRR. Annual Report 2016; 2016.
